# Trends in emergency contraception awareness among women and girls in 28 sub-Saharan countries

**DOI:** 10.1186/s12889-021-12067-y

**Published:** 2021-11-03

**Authors:** Oluwafemi Emmanuel Awopegba, Okechukwu Stephen Chukwudeh, Eyitayo Omolara Owolabi, Anthony Idowu Ajayi

**Affiliations:** 1 Economics and Business Policy Department, Nigerian Institute of Social and Economic Research, Ibadan, Nigeria; 2 Department of Criminology and Security Studies, Faculty of Social Sciences, Federal University, Oye-Ekiti, Nigeria; 3 Centre for Global Surgery, Department of Global Health, Stellenbosch University, Cape Town, South Africa; 4 Population Dynamics and Sexual and Reproductive Health, African Population and Health Research Centre, APHRC Campus, Manga Close, Nairobi, Kenya

**Keywords:** Awareness, Emergency contraception, Sub-Saharan Africa, Trends, Unintended pregnancy

## Abstract

**Background:**

Studies have shown that emergency contraception (EC) remains underutilised in preventing unintended pregnancy in sub-Saharan Africa (SSA). Small-scale surveys have attributed EC underutilisation to gaps in EC awareness among SSA women and girls. However, limited studies have explored trends in EC awareness in SSA. We address this gap by examining trends in EC awareness using data from 28 SSA countries. Our analysis was disaggregated by age distribution, place of residence, level of education, and wealth to show differences in EC awareness trend.

**Methods:**

We analysed the Demographic and Health Surveys (DHS) data of 1,030,029 women aged 15 to 49 on emergency contraception awareness. EC awareness was defined as having ever heard of special pills to prevent pregnancy within 3 days after unprotected sexual intercourse. Frequencies and percentages were used to summarise trends in EC awareness between years 2000 and 2019.

**Results:**

Overall, there was an upward shift in the level of EC awareness in all countries, except in Burkina Faso, Niger, Chad, and Ethiopia. While some countries recorded a significant upward trend in EC awareness, others recorded just a slight increase. Women in Kenya, Ghana, Gabon, and Cameroon had the highest upward shift in EC awareness. For example, only 28% of women were aware of EC in Ghana in 2003, but in 2014, 64% of women knew about EC, an increase of over 36 percentage points. Increase in EC awareness was starker among women aged 20–24 years, those who resided in urban areas, had higher education, and belong to the highest wealth quintile, than those aged 15–19, in rural areas, with no formal education and belonging to the lowest wealth quintile.

**Conclusion:**

Our analysis shows that the level of EC awareness has increased substantially in most SSA countries. However, EC awareness still differs widely within and between SSA countries. Intervention to improve EC awareness should focus on women aged 15 to 19, those with no formal education, residing in rural areas, and within the lowest quintile, especially, in countries such as Chad, Niger, Burkina Faso, and Ethiopia where level of EC is low with lagging progress.

**Supplementary Information:**

The online version contains supplementary material available at 10.1186/s12889-021-12067-y.

## Introduction

Unplanned pregnancy and unsafe abortion are global social problems with severe socioeconomic, health, and demographic consequences. Despite conscientious efforts by public health experts, governments, and non-governmental organizations, particularly since the 1994 International Conference on Population and Development (ICPD) in Cairo, the prevalence of unplanned pregnancies has barely declined [1]. A recent study shows that 49% of approximately 111 million pregnancies in Low and Middle-Income Countries (LMIC) are unplanned [2]. Notably, most pregnancies among adolescent girls are unplanned [3]. An estimated 42% of pregnancies in sub-Saharan Africa are unintended [2].

Unintended pregnancy is the main reason women and girls seek abortion. When women experience unintended pregnancy, some carry it to term while others seek abortion services. In SSA, about 37% of women with unintended pregnancies terminate it. Due to restrictive abortion laws, most women (77%) seek abortion from untrained persons or providers using non-recommended methods or both, resulting in complications, hospitalisation, disabilities, and deaths [4]. An estimated 6.2 million unsafe abortions occur each year in SSA, and 1.6 million women are treated for unsafe abortion-related complications [1]. The highest abortion-related deaths occur in SSA, and an estimated 50% of these deaths occur among young people [5].

Unintended pregnancy and unsafe abortion could be prevented with the use of contraceptives, especially emergency contraception (EC) [6, 7]. However, the use of all contraceptive methods, particularly EC, remains low in most sub-Saharan Africa countries [8, 9]. EC is effective in preventing pregnancy after sex [7]. Women who engage in unprotected sex voluntarily or involuntarily could still prevent unintended pregnancy if they are aware and can access emergency contraception. EC is available over the counter, without prescription, and has existed for more than four decades [9]. Its continued underutilisation suggests women and girls lack knowledge of its availability and benefits.

Previous studies—although primarily small-scale surveys and among young women—have shown that just over two in three young women are aware of EC, and a little over half of them have the correct knowledge [10–18]. While many were not aware of emergency contraception, others lack knowledge of the correct EC pills and timing of use [10,11]. In addition, some women were misinformed about its side effects, erroneously linking it to infertility [11]. The lack of comprehensive sexuality education contributes to the awareness gaps on EC in sub-Saharan Africa. In general, there has been more attention to programmes promoting access to contraceptive information and services over the past two decades. However, the extent to which awareness of emergency contraception has increased among women and girls over the past 20 years (2000–2020) is unknown. Our study fills this gap by examining the trends of EC awareness in sub-Saharan Africa, using data from 28 countries. In addition, we examined the trends in EC awareness by age distribution, place of residence, level of education, and wealth.

## Methods and materials

We analysed the Demographic and Health Surveys (DHS) data of 1,030,029 women aged 15 to 49 on emergency contraception awareness. The DHS is a nationally representative survey collected every 5 years across low- and middle-income countries. Given the objective of the study is to investigate trends of EC among women in SSA, we limited the scope to 28 countries in SSA that have two survey years information on EC awareness. Following the DHS guidelines, weightings were applied to obtain unbiased estimates. Bias can result in oversampled sub-populations by not using weights. A weighted sample of 1,030,151 women were considered. The list of countries, survey years, and analytic samples are provided in Table 1. Details on the data collection and sampling methodology used by the DHS can be accessed elsewhere [19].

**Table 1:** Country and year sample

Country	DHS Year	Unweighted Sample	Weighted Sample
SSA	–	1,030,029	1,030,151
Western			
Benin	2001	6210	6210
	2006	17,676	17,681
	2012	16,599	16,599
	2018	15,928	15,928
Burkina Faso	2003	12,472	12,471
	2010	17,072	17,070
Gambia	2011	10,202	10,209
	2020	11,865	11,865
Ghana	2003	5685	5688
	2008	4907	4907
	2014	9390	9393
Guinea	2005	7925	7925
	2012	9128	9124
	2018	10,874	10,874
Liberia	2007	7056	7061
	2013	9210	9206
	2019	8065	8065
Mali	2001	12,828	12,829
	2006	14,538	14,539
	2012	10,424	10,424
	2018	10,519	10,519
Niger	2006	9201	9205
	2012	11,138	11,137
Nigeria	2003	7590	7581
	2008	33,243	33,240
	2013	38,823	38,820
	2018	41,821	41,821
Senegal	2005	14,570	14,570
	2011	15,688	15,688
	2014	8488	8488
	2019	8649	8649
Sierra Leone	2008	7360	7359
	2013	16,630	16,627
	2019	15,574	15,574
Central			
Burundi	2010	9379	9376
	2016	17,269	17,269
Cameroon	2004	10,637	10,635
	2011	15,390	15,386
	2018	13,527	13,616
Chad	2004	6084	6085
	2015	17,661	17,663
Congo	2005	7003	39
	2011	10,790	100
Congo DRC	2007	9969	9966
	2013	18,779	18,793
Gabon	2001	6167	6170
	2012	8356	8358
Eastern			
Ethiopia	2011	16,497	16,507
	2016	15,683	15,683
Kenya	2003	8189	8189
	2009	8442	8443
	2014	31,072	31,075
Madagascar	2004	7947	7949
	2009	17,364	17,368
Rwanda	2000	10,412	10,410
	2005	11,302	11,302
	2010	13,664	13,664
	2015	13,489	13,486
Tanzania	2004	10,317	10,318
	2010	10,135	10,135
	2015	13,265	13,265
Uganda	2006	8524	8525
	2011	8669	8671
	2016	18,506	18,506
Southern			
Lesotho	2004	7094	7095
	2009	7624	7624
	2014	6621	6621
Malawi	2000	13,189	13,184
	2004	11,689	11,685
	2010	23,003	23,006
	2015	24,562	24,562
Namibia	2000	6745	6748
	2007	9784	9785
	2013	9164	9168
Zambia	2002	7644	7643
	2007	7146	7146
	2013	16,325	16,330
	2018	13,683	13,683
Zimbabwe	1999	5899	5901
	2005	8895	8895
	2010	9171	9171
	2015	9955	9955

### Study variables

The main variable of interest is the awareness of EC method, measured as “Yes” if the respondent knows about EC and “No if the respondent does not know about EC. We disaggregated the women by 5-year age groups, place of residence, wealth status, and education level to explore socio-demographic differences. Age groups comprised of ages 15 to 19 (adolescents), 20 to 24 (young adults) and 25 to 49 (adults). Residence was divided into “Rural” and “Urban” areas. Wealth status was assessed as an index of household assets and utilities, and categorized as “Poorest” “Poorer”, “Middle”, “Richer,” and “Richest”. Educational level was classified as “No formal education”, “Primary”, “Secondary and Higher”.

### Statistical analysis

To calculate the proportion of young women who knew of EC, we used descriptive statistics. The proportions for each SSA country are presented as percentages in a panel line graph format. Next, we present the association between EC and socio-demographic factors such as age, place of residence, wealth status, and education level, using Pearson’s chi-square test. We carried out the analyses using STATA version 16.0.

### Ethical considerations

This study only analysed de-identified publicly available data obtained in line with the highest ethical standard for conducting human subject research; therefore, we did not seek ethical approval. The DHS surveys are conducted after approval of ethical review bodies and authorization by the country of the study. De-identified datasets are freely available on the DHS website (https://dhsprogram.com/data/available-datasets.cfm).

## Results

### Trends in EC awareness within and between countries

**Fig. 1:**
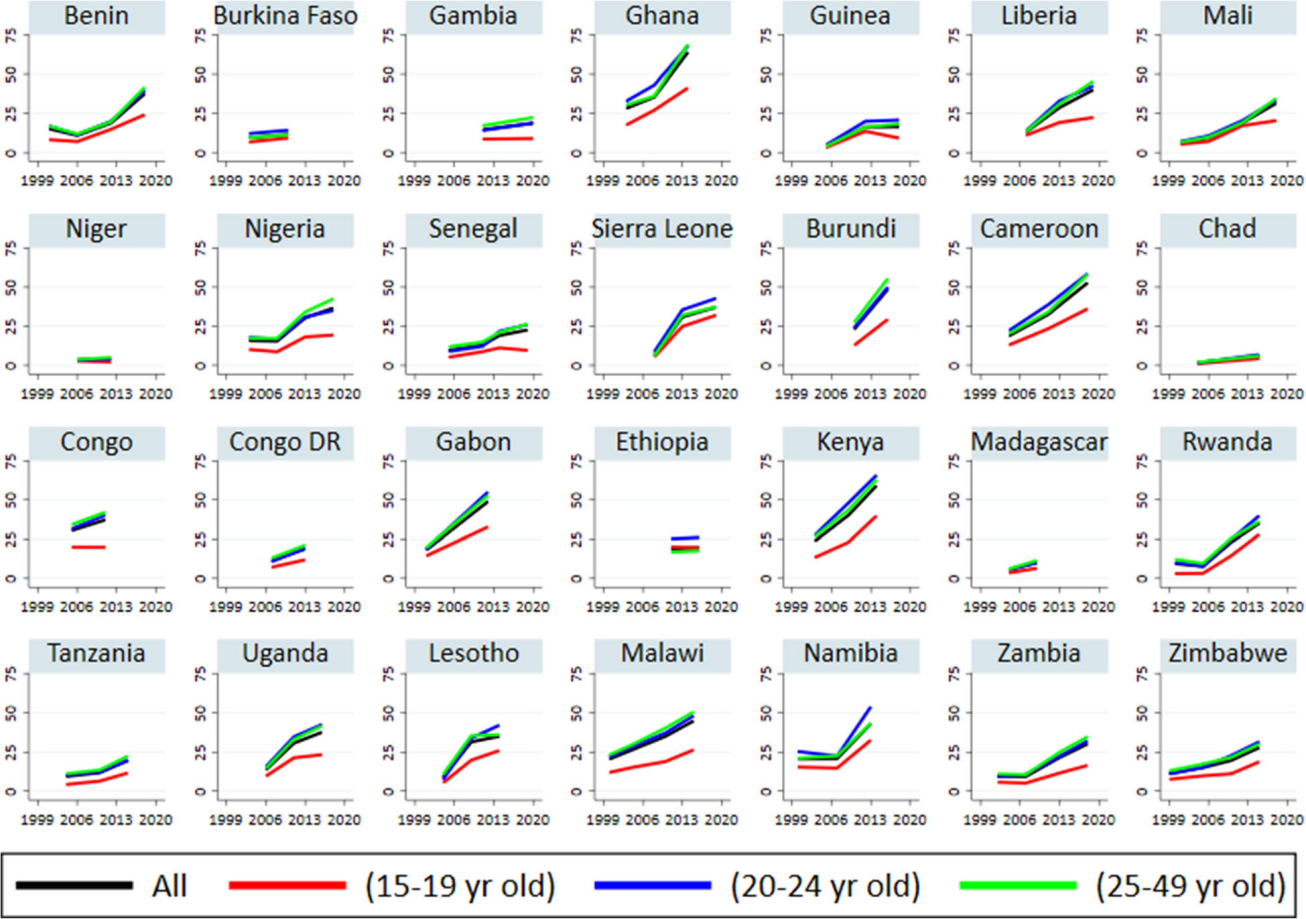
Trends in EC awareness by age categories (15 to 19, 20 to 24 and 25 to 49 year) in SSA between 1999 and 2019

Figure 1 depicts the trends in women’s awareness of emergency contraception (EC) in 28 SSA countries. While over half of women in Ghana (64%), Kenya (59%), and Cameroon (53%) were aware of EC, only one in ten women knew of EC in Chad (5.8%), Niger (4.4%), Madagascar (10.1%) and Burkina Faso (11.6%). Overall, there was an upward shift in the level of EC awareness in all countries, except in Burkina Faso, Niger, Chad, and Ethiopia. While some countries recorded a significant upward trend in EC awareness, others recorded just a slight increase. Women in Kenya, Ghana, Gabon, and Cameroon had the highest upward shift in EC awareness. For example, only 28% of women were aware of EC in Ghana in 2003, but in 2014, 64% of women knew about EC, an increase of over 36 percentage points.

The level of awareness of EC is lowest among adolescent girls (15–19 years) in all countries studied. In Ghana, for example, the difference in EC awareness between adolescent girls (15–19 years) and young adults (20–24 years) was approximately 27 percentage points in 2014. We observed an upward trend in EC awareness in all age groups in most countries studied. However, it appears that the upward trend is steeper in the age group 20–24 years than in other age categories.

### Urban and rural trends

**Fig. 2:**
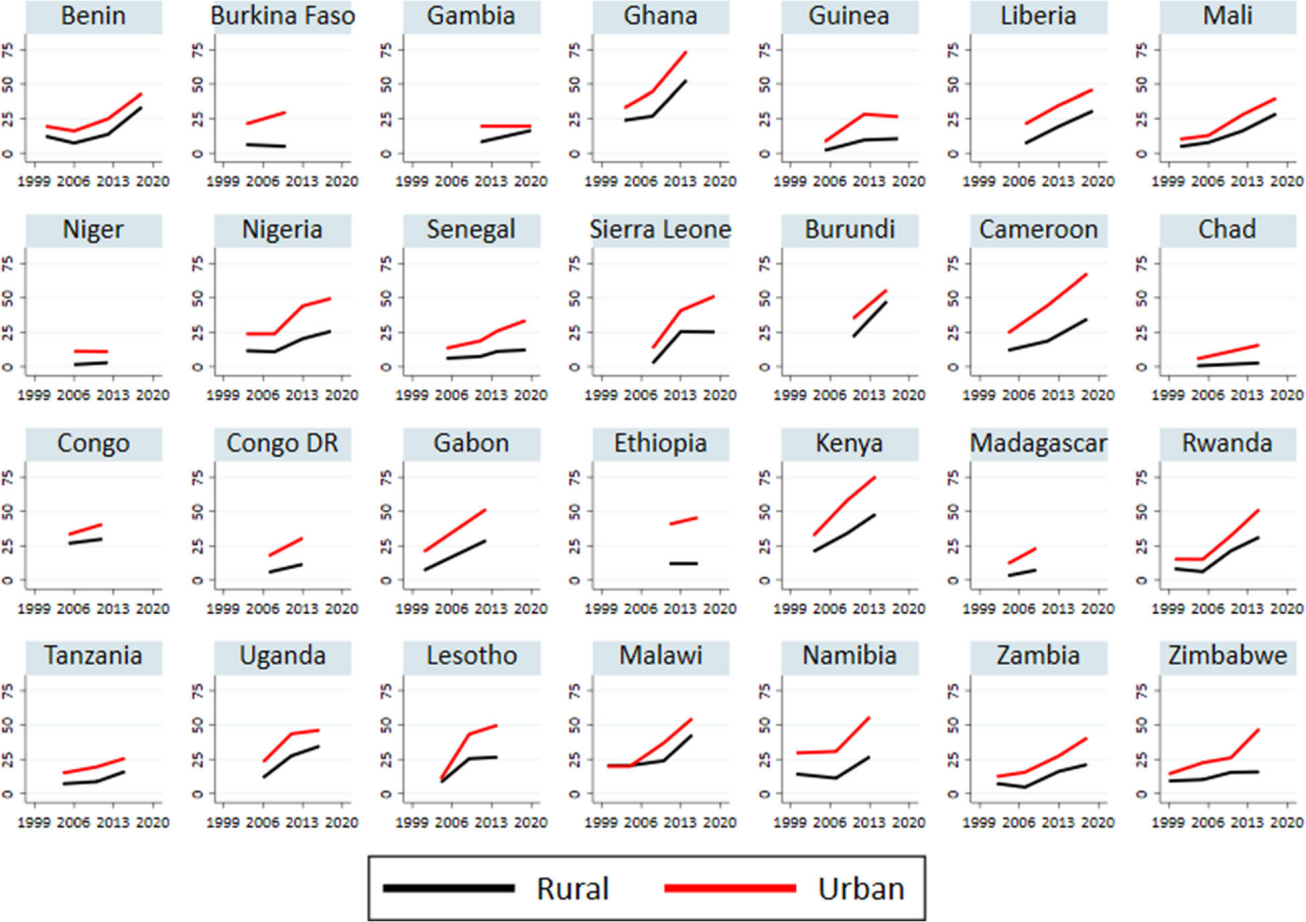
Trends in EC awareness by place of residence in SSA between 1999 and 2019

Figure 2 illustrates the proportion of women of reproductive age who knew about EC by place of residence. EC awareness was significantly higher among women residing in urban areas than those in rural areas in all countries studied. While EC awareness has increased in both rural and urban areas in most countries, the upward trend is far steeper for urban areas than in rural areas. For example, EC awareness rose by 40 percentage points (from 32.8% in 2004 to 73.8% in 2014) in urban areas in Ghana compared to 20% in rural areas (from 23.8% in 2004 to 52.9% in 2014). Similarly, EC awareness rose by 43% in Cameroon and Kenya in urban areas compared to 13% in rural areas in Cameroon and 28% in rural Kenya. However, in countries like Niger, Ethiopia, Chad, and Burkina Faso, the level of awareness of EC remains unchanged among rural women.

### Trends by level of education

**Fig. 3:**
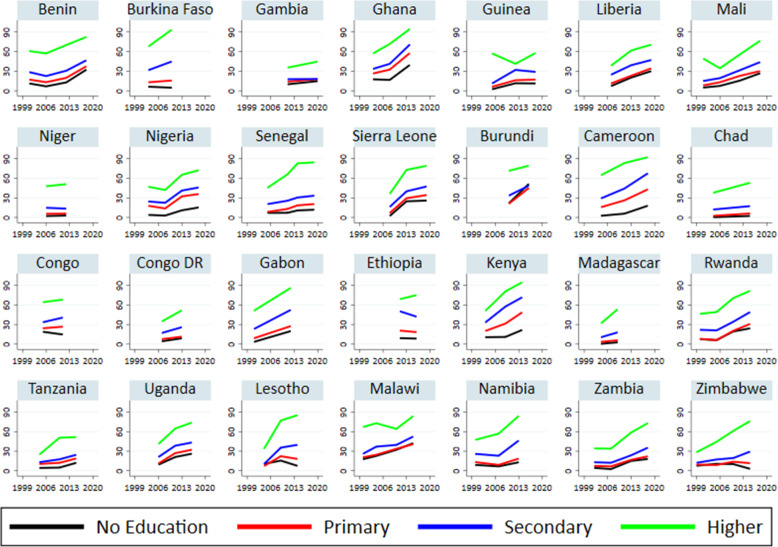
Trends in EC awareness by education level in 28 sub-Saharan countries between 1999 and 2019

Trend in the proportion of women who knew about EC by their level of education is depicted in Fig. 3. The magnitude of variation in EC awareness by levels of education is stark. While four in five women with higher education were aware of EC in 15 of the 28 countries studied, only about one in ten women with no education knew of EC in 15 countries, one in five in three countries (Cameroon, Kenya, and Gabon), two in five in two countries (Ghana and Malawi), and one in three in Benin and Liberia. There was an upward trend in EC awareness for all education categories, except in nine countries (Burkina Faso, Niger, Congo, Ethiopia, Congo Dr., Chad, Madagascar, Zimbabwe, and Lesotho), where EC awareness remained unchanged among women with no formal education. The increase in EC awareness is starker among women with higher education than for women with other educational levels.

### Trends by wealth index

**Fig. 4:**
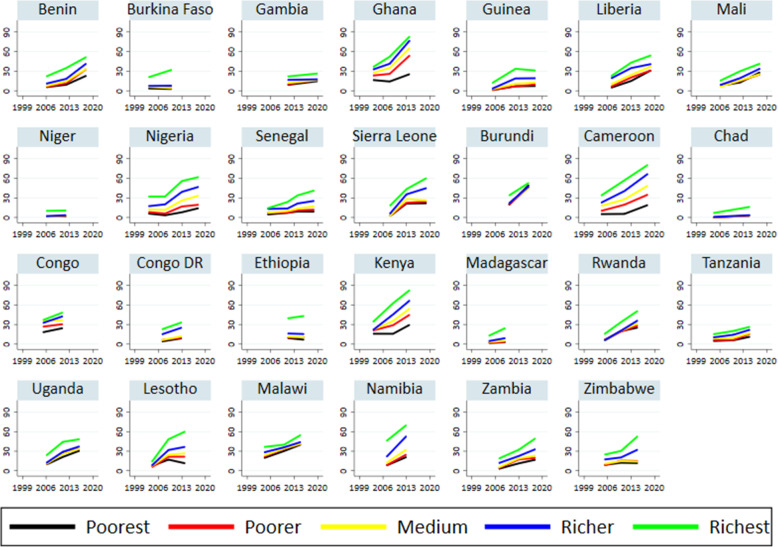
Trends in EC awareness by wealth status in 28 sub-Saharan countries: 1999 to 2019

Figure 4 depicts trends in EC awareness by wealth quintiles in 28 sub-Saharan African countries. Generally, EC awareness differs substantially by wealth status in all countries studied. Women from the poorest households were the least aware of EC than women from the richest households. For example, more than one in two women in the richest wealth quintile knew of EC in 14 of the 28 countries studied relative to only one in ten in 11 countries. Also, EC awareness improved more substantially among women in the wealthiest quintile than among women in the lowest wealth quintile. For example, EC awareness increased among women in the richest wealth quintile by 50% in Ghana, 43% in Sierra Leone, 47% in Cameroon, 50% in Kenya, and 47% in Lesotho, relative to only 9% among women in the poorest households in Ghana, 20% in Sierra Leone, 14% in Cameroon, 15% in Kenya, and 4% in Lesotho.

## Discussion

Emergency contraception can prevent unplanned pregnancy after unprotected sex or rape, but gaps exist in women’s awareness of this method. We conducted a trend analysis of women’s awareness of EC using the DHS data of 28 SSA countries. Our analyses show that EC awareness significantly increased in most countries between the years 2000 and 2019. However, a few countries recorded no progress. The increase in EC awareness is not surprising given the increased focus on improving women’s knowledge of contraceptives in SSA [20] since the 1994 International Conference on Population and Development. Many development partners and governments have implemented programmes to boost women’s knowledge and use of contraceptives. What is surprising is the lack of progress in Chad, Niger, Burkina Faso, and Ethiopia despite efforts to improve women’s EC awareness in these countries. It is plausible the family planning programmes in these countries have not prioritised emergency contraceptives.

One notable finding of this study is that increase in EC awareness remain uneven within countries. While EC awareness has increased substantially among young women aged 20 to 24, a slight improvement was recorded among girls aged 15–19 in most countries. Girls aged 15–19 have relatively low awareness of EC compared to young women aged 20–24 in all countries studied. The differences could be explained by better exposure of young women (aged 20–24) to information sources relative to adolescent girls [21–23]. In most SSA countries, sexuality education is abstinence-based and HIV prevention-focused, neglecting aspects like contraceptive methods, including EC [24]. However, as young people initiate sex, their need for contraceptive information would increase. Because of the higher prevalence of sexual activities among females aged 20–24 years than 15–19 years old, they will likely source for information on the subject more than the adolescent girls. Health care visit for sexual and reproductive health services is higher among young women aged 20–24 years than adolescent girls [25], thus, increasing their chances of learning about EC. Improvement in EC awareness among adolescent girls would remain slow, except they are deliberately targeted with such information through sexuality education programmes.

We also found huge rural and urban differences in EC awareness in most countries. Increase in EC awareness was substantial in urban areas than in rural areas in all countries studied. This finding is expected given that women in urban areas have better access to the Internet, media platforms, and health providers than women in rural areas in most SSA countries [21, 23, 25]. Access to the Internet grew rapidly between 2000 and 2019 in SSA [26], and this could have helped many women access EC information without needing to consult health providers. In several SSA countries, access to electricity, the Internet, and smartphones in rural areas remain limited compared to urban areas. As a result, women in rural areas are less likely to learn about EC through the Internet. Another plausible reason for the rural and urban differences in EC awareness progress is that family planning programmes targeting improving access to information and services are implemented in urban areas than rural areas. Social marketing of family planning methods is widely available in urban areas than rural areas [27], which could explain the wide rural and urban differences in EC awareness.

We also observed a stark increase in EC awareness among women with higher education than those with lower educational levels in all countries. Similar finding was observed when we stratified our analysis by wealth status. Much of the increase in EC awareness in SSA occurred among women with a higher level of education and those in the highest wealth quintile. This is not surprising given that they tend to have access to information from multiple sources, including the Internet and through their social networks, compared to less educated women and those in the lowest wealth quintile. However, the slow increase in EC awareness among less-educated women and those in the lowest wealth quintile is concerning, given the importance of the knowledge of unplanned pregnancy prevention. It means they may become susceptible to unintended pregnancy and potentially carry such an unwanted pregnancy to term because abortion services are restricted in most SSA settings. In addition, low EC awareness among less-educated women is one reason for the underutilisation of EC and its limited effect in significantly reducing unintended pregnancy in SSA.

### Study strengths and limitations

The definition of EC as special pills women can use to prevent pregnancy is a limitation of our study, given that studies have shown that a few women considered non-EC drugs and concoctions as EC [10, 11]. This limitation could have slightly inflated the proportion of women that were aware of the correct EC methods. However, our use of large datasets from 28 SSA countries is important strength of this study, allowing us to report robust findings on differing EC awareness within and between SSA countries.

## Conclusion

Our analysis shows that the level of EC awareness has increased substantially in most SSA countries. However, EC awareness still differs significantly within and between SSA countries. In addition, increase in EC awareness was starker among women aged 20–24 years, who resided in urban areas, had higher education, and belonged to the highest wealth quintile. Intervention to improve EC awareness should focus on countries like Chad, Niger, Burkina Faso, and Ethiopia, where awareness remained low and minimal progress was recorded.

## Supplementary Information

**Additional file 1: Table S1.** Prevalence of EC and Association between EC and Socio-Demographics of women in 28 sub-Saharan countries between 1999 and 2019.

## Data Availability

The DHS data utilised for this study is publicly available via https://www.dhsprogram.com/data/available-datasets.cfm

## Abbreviations

EC: Emergency contraception
SSA: Sub-Saharan Africa
DHS: Demographic and Health Surveys
LMICs: Low-and middle-income countries
HIV: Human Immunodeficiency Virus
