# Supplementary material for: Trends in emergency contraception awareness among women and girls in 28 sub-Saharan countries
Source: BMC Public Health. 2021 Nov 3;21:1987. doi: 10.1186/s12889-021-12067-y (PMC8567620; doi:10.1186/s12889-021-12067-y)
Supplement: Supplementary file 1 — Additional file 1: Table S1. Prevalence of EC and Association between EC and Socio-Demographics of women in 28 sub-Saharan countries between 1999 and 2019. [file 12889_2021_12067_MOESM1_ESM.docx]

Table S1: Prevalence of EC and Association between EC and Socio-Demographics of women in 28 sub-Saharan countries between 1999 and 2019

| **Country** | **Year** | **All** | **15-19** | **20-24** | **25-49** | **Rural** | **Urban** | **No Edu-cation** | **Pri- mary** | **Second- ary** | **Higher** | **poorest** | **poorer** | **middle** | **richer** | **richest** |
| --- | --- | --- | --- | --- | --- | --- | --- | --- | --- | --- | --- | --- | --- | --- | --- | --- |
| Western |  |  |  |  |  |  |  |  |  |  |  |  |  |  |  |  |
| Benin | 2001 | 15.2 | 8.3 | 17.0 | 16.9 | 12.3 | 19.5 | 11.2 | 17.2 | 28.6 | 60.9 |  |  |  |  |  |
|  | 2006 | 11.0 | 7.0 | 11.4 | 11.9 | 7.3 | 16.1 | 6.8 | 13.1 | 22.5 | 57.2 | 5.5 | 6.0 | 7.3 | 10.8 | 21.8 |
|  | 2012 | 18.9 | 14.9 | 20.0 | 19.7 | 13.6 | 24.9 | 12.8 | 19.8 | 30.7 | 70.0 | 9.3 | 11.5 | 14.5 | 18.3 | 34.9 |
|  | 2018 | 37.7 | 24.2 | 39.8 | 41.8 | 33.5 | 43.4 | 32.4 | 37.5 | 46.7 | 82.5 | 23.1 | 33.4 | 33.7 | 41.8 | 51.8 |
| Burkina Faso | 2003 | 9.4 | 6.7 | 12.1 | 9.5 | 6.1 | 21.2 | 6.2 | 13.0 | 31.5 | 67.6 | 3.6 | 6.7 | 6.0 | 7.3 | 20.5 |
|  | 2010 | 11.6 | 9.3 | 14.3 | 11.5 | 4.9 | 29.7 | 4.7 | 15.7 | 44.9 | 93.8 | 2.8 | 4.2 | 4.1 | 7.8 | 32.2 |
| Gambia | 2011 | 14.6 | 8.6 | 14.1 | 17.2 | 8.1 | 19.6 | 9.9 | 14.1 | 17.7 | 35.4 | 9.0 | 10.0 | 12.0 | 16.7 | 21.8 |
|  | 2020 | 18.8 | 9.0 | 18.9 | 22.4 | 16.5 | 19.6 | 14.8 | 17.7 | 18.0 | 44.7 | 14.7 | 16.8 | 16.4 | 17.5 | 26.4 |
| Ghana | 2003 | 28.2 | 17.5 | 32.8 | 30.3 | 23.8 | 32.8 | 17.6 | 26.4 | 33.5 | 57.0 | 16.4 | 23.3 | 27.2 | 32.5 | 36.2 |
|  | 2008 | 35.5 | 27.1 | 43.1 | 36.1 | 26.9 | 44.6 | 16.6 | 32.4 | 41.3 | 71.7 | 14.2 | 25.9 | 35.0 | 41.5 | 52.2 |
|  | 2014 | 64.1 | 41.4 | 68.0 | 69.1 | 52.9 | 73.8 | 39.6 | 57.8 | 70.9 | 94.8 | 25.6 | 54.4 | 65.8 | 77.4 | 83.6 |
| Guinea | 2005 | 3.9 | 3.2 | 4.8 | 4.0 | 2.0 | 8.3 | 2.3 | 6.0 | 11.0 | 57.3 | 1.0 | 1.3 | 2.3 | 3.0 | 11.6 |
|  | 2012 | 16.4 | 13.6 | 20.0 | 16.3 | 9.6 | 28.3 | 11.5 | 16.1 | 32.0 | 41.3 | 6.9 | 6.9 | 10.8 | 18.9 | 33.6 |
|  | 2018 | 16.5 | 9.2 | 20.8 | 18.3 | 10.5 | 26.5 | 11.2 | 17.2 | 28.9 | 57.9 | 7.4 | 9.7 | 12.5 | 19.2 | 31.0 |
| Liberia | 2007 | 13.0 | 11.0 | 13.6 | 13.3 | 7.0 | 21.1 | 7.1 | 11.1 | 24.6 | 38.3 | 4.7 | 6.2 | 9.8 | 18.8 | 22.2 |
|  | 2013 | 28.8 | 19.2 | 33.1 | 31.1 | 19.4 | 34.8 | 20.2 | 22.9 | 39.1 | 61.6 | 15.0 | 20.3 | 24.3 | 34.7 | 43.1 |
|  | 2019 | 40.2 | 22.4 | 42.6 | 45.6 | 30.6 | 46.1 | 30.1 | 34.3 | 47.1 | 70.7 | 31.6 | 31.4 | 37.5 | 41.0 | 54.4 |
| Mali | 2001 | 6.4 | 5.3 | 7.0 | 6.5 | 4.8 | 10.1 | 4.9 | 8.2 | 14.9 | 49.5 |  |  |  |  |  |
|  | 2006 | 9.4 | 7.1 | 10.7 | 9.8 | 7.7 | 12.8 | 7.4 | 13.0 | 19.4 | 34.5 | 7.9 | 7.7 | 6.1 | 8.7 | 15.2 |
|  | 2012 | 19.0 | 17.2 | 20.4 | 19.1 | 16.1 | 27.8 | 15.7 | 22.9 | 31.6 | 55.9 | 12.7 | 15.0 | 15.6 | 19.4 | 29.9 |
|  | 2018 | 31.6 | 20.4 | 33.5 | 34.6 | 28.6 | 40.0 | 26.8 | 30.1 | 43.9 | 76.7 | 28.5 | 25.3 | 25.6 | 34.1 | 41.7 |
| Niger | 2006 | 3.4 | 2.8 | 3.3 | 3.7 | 1.5 | 11.3 | 2.2 | 5.5 | 14.8 | 47.7 | 1.8 | 1.3 | 1.3 | 1.9 | 10.0 |
|  | 2012 | 4.4 | 2.2 | 4.0 | 5.0 | 2.9 | 10.9 | 2.9 | 5.8 | 13.5 | 51.0 | 1.6 | 2.4 | 2.6 | 3.5 | 10.7 |
| Nigeria | 2003 | 15.8 | 10.1 | 17.8 | 17.3 | 11.6 | 23.8 | 3.9 | 17.9 | 24.4 | 47.0 | 6.0 | 8.3 | 12.3 | 17.2 | 32.0 |
|  | 2008 | 15.5 | 8.7 | 16.7 | 17.2 | 10.8 | 23.8 | 2.8 | 13.7 | 22.4 | 42.0 | 3.4 | 5.8 | 11.5 | 20.1 | 32.1 |
|  | 2013 | 30.4 | 18.1 | 31.0 | 34.1 | 20.3 | 44.2 | 10.9 | 32.3 | 41.1 | 65.3 | 7.9 | 16.7 | 25.8 | 39.2 | 55.7 |
|  | 2018 | 36.7 | 19.3 | 35.0 | 42.6 | 25.7 | 49.7 | 15.3 | 35.8 | 45.9 | 72.3 | 14.3 | 19.4 | 33.4 | 46.9 | 61.9 |
| Senegal | 2005 | 9.7 | 5.2 | 8.8 | 11.9 | 6.1 | 13.4 | 7.0 | 8.4 | 20.4 | 45.3 | 4.6 | 7.1 | 7.0 | 13.1 | 14.0 |
|  | 2011 | 13.0 | 8.7 | 12.2 | 14.9 | 7.4 | 18.8 | 7.1 | 13.1 | 25.6 | 66.0 | 7.1 | 7.2 | 9.8 | 13.6 | 23.7 |
|  | 2014 | 19.1 | 11.2 | 21.7 | 21.1 | 11.1 | 26.0 | 10.8 | 18.5 | 30.5 | 82.8 | 9.1 | 10.8 | 13.3 | 21.3 | 33.7 |
|  | 2019 | 22.7 | 9.6 | 26.1 | 26.4 | 12.1 | 33.6 | 11.8 | 20.5 | 33.3 | 84.4 | 8.9 | 11.9 | 17.2 | 25.4 | 41.3 |
| Sierra Leone | 2008 | 6.2 | 5.2 | 8.5 | 5.9 | 2.3 | 13.3 | 2.2 | 6.8 | 16.1 | 36.0 | 1.8 | 2.0 | 1.9 | 5.3 | 17.6 |
|  | 2013 | 31.0 | 24.9 | 35.5 | 32.1 | 25.5 | 40.9 | 24.7 | 29.4 | 40.0 | 72.9 | 21.3 | 22.4 | 28.3 | 35.4 | 43.4 |
|  | 2019 | 37.2 | 32.0 | 42.8 | 37.5 | 25.2 | 51.3 | 25.9 | 34.3 | 47.5 | 79.1 | 21.7 | 24.3 | 26.1 | 45.1 | 60.4 |
| Central |  |  |  |  |  |  |  |  |  |  |  |  |  |  |  |  |
| Burundi | 2010 | 23.0 | 12.7 | 23.9 | 27.3 | 21.6 | 34.9 | 21.4 | 20.9 | 33.3 | 71.4 | 18.8 | 19.6 | 21.8 | 21.2 | 33.3 |
|  | 2016 | 48.7 | 29.5 | 50.1 | 55.5 | 47.6 | 56.0 | 51.5 | 45.2 | 48.6 | 79.3 | 50.0 | 46.4 | 45.9 | 47.9 | 53.1 |
| Cameroon | 2004 | 18.8 | 12.9 | 22.1 | 20.2 | 11.9 | 24.4 | 2.5 | 15.7 | 29.1 | 64.6 | 5.1 | 9.9 | 17.6 | 22.4 | 33.2 |
|  | 2011 | 32.6 | 23.4 | 38.8 | 34.1 | 18.6 | 44.5 | 5.8 | 26.2 | 44.2 | 83.3 | 5.4 | 19.5 | 27.5 | 40.4 | 56.8 |
|  | 2018 | 52.9 | 36.1 | 58.6 | 58.1 | 34.6 | 67.6 | 18.0 | 43.0 | 67.7 | 92.3 | 19.1 | 35.0 | 48.9 | 66.9 | 80.7 |
| Chad | 2004 | 1.6 | 1.0 | 1.5 | 1.9 | 0.6 | 5.6 | 0.4 | 2.4 | 12.1 | 37.8 | 0.0 | 0.2 | 0.7 | 0.7 | 6.6 |
|  | 2015 | 5.8 | 4.3 | 6.8 | 6.0 | 2.7 | 15.7 | 2.3 | 6.1 | 17.3 | 53.1 | 2.7 | 2.4 | 2.9 | 3.4 | 16.3 |
| Congo | 2005 | 30.4 | 19.9 | 31.5 | 34.2 | 26.8 | 33.3 | 18.9 | 24.2 | 33.5 | 64.2 | 18.1 | 26.7 | 35.9 | 32.3 | 36.7 |
|  | 2011 | 37.3 | 19.8 | 40.3 | 42.1 | 29.9 | 40.7 | 14.7 | 26.7 | 40.7 | 68.2 | 24.5 | 30.6 | 36.8 | 42.7 | 48.5 |
| Congo DR | 2007 | 11.2 | 7.1 | 10.7 | 12.9 | 5.8 | 17.7 | 4.3 | 7.6 | 17.0 | 34.6 | 4.1 | 6.2 | 6.2 | 14.9 | 22.5 |
|  | 2013 | 18.8 | 11.9 | 19.4 | 21.2 | 11.5 | 30.6 | 8.9 | 11.3 | 25.9 | 51.8 | 8.9 | 10.0 | 12.0 | 25.3 | 33.4 |
| Gabon | 2001 | 18.1 | 14.4 | 18.6 | 19.6 | 7.2 | 20.7 | 3.4 | 8.9 | 23.1 | 51.1 |  |  |  |  |  |
|  | 2012 | 49.1 | 32.8 | 55.2 | 52.9 | 28.9 | 51.7 | 20.0 | 27.6 | 52.5 | 86.2 |  |  |  |  |  |
| Eastern |  |  |  |  |  |  |  |  |  |  |  |  |  |  |  |  |
| Ethiopia | 2011 | 19.0 | 20.0 | 25.1 | 16.7 | 12.1 | 40.7 | 9.2 | 20.7 | 50.3 | 68.9 | 9.3 | 10.1 | 11.6 | 16.3 | 39.4 |
|  | 2016 | 19.5 | 19.5 | 26.0 | 17.6 | 12.1 | 45.6 | 8.4 | 18.3 | 42.0 | 75.1 | 6.7 | 10.6 | 10.3 | 15.4 | 43.2 |
| Kenya | 2003 | 23.7 | 13.3 | 27.3 | 26.6 | 20.8 | 32.4 | 10.6 | 20.1 | 33.0 | 50.7 | 15.9 | 20.8 | 21.9 | 21.4 | 33.7 |
|  | 2009 | 40.2 | 23.0 | 48.1 | 43.6 | 34.1 | 58.1 | 11.0 | 31.4 | 57.5 | 80.7 | 15.8 | 28.9 | 35.4 | 45.4 | 62.6 |
|  | 2014 | 59.2 | 39.9 | 65.9 | 63.0 | 48.0 | 75.4 | 21.7 | 48.7 | 71.7 | 94.7 | 29.8 | 45.2 | 54.6 | 67.1 | 83.0 |
| Madagascar | 2004 | 5.5 | 3.5 | 5.7 | 6.0 | 3.3 | 12.1 | 0.5 | 3.5 | 10.4 | 31.7 | 1.8 | 2.3 | 3.3 | 4.6 | 12.5 |
|  | 2009 | 10.1 | 6.5 | 10.1 | 11.5 | 7.4 | 23.2 | 2.7 | 5.8 | 18.0 | 53.5 | 3.0 | 3.5 | 5.2 | 9.1 | 24.7 |
| Rwanda | 2000 | 9.5 | 3.1 | 10.2 | 12.1 | 8.3 | 15.3 | 7.6 | 8.0 | 22.0 | 46.4 |  |  |  |  |  |
|  | 2005 | 7.7 | 3.2 | 7.7 | 9.6 | 6.2 | 15.2 | 6.1 | 6.2 | 21.0 | 49.1 | 5.3 | 6.5 | 5.6 | 5.7 | 15.1 |
|  | 2010 | 23.0 | 14.5 | 24.8 | 25.6 | 21.4 | 32.4 | 19.4 | 20.4 | 34.3 | 70.6 | 19.9 | 19.3 | 20.5 | 20.6 | 33.7 |
|  | 2015 | 35.2 | 28.1 | 40.0 | 36.2 | 31.3 | 51.5 | 24.2 | 31.0 | 49.1 | 81.6 | 25.5 | 28.5 | 31.9 | 36.4 | 51.0 |
| Tanzania | 2004 | 9.4 | 4.2 | 10.2 | 11.1 | 7.2 | 15.0 | 4.4 | 10.5 | 13.1 | 24.8 | 4.8 | 6.3 | 8.6 | 10.3 | 15.1 |
|  | 2010 | 11.8 | 6.4 | 12.4 | 13.6 | 8.8 | 19.5 | 4.9 | 12.2 | 17.6 | 50.6 | 5.9 | 6.4 | 9.5 | 14.3 | 20.1 |
|  | 2015 | 19.6 | 11.6 | 19.9 | 22.5 | 16.1 | 25.8 | 12.3 | 18.9 | 24.5 | 51.6 | 11.4 | 16.1 | 17.0 | 22.2 | 26.6 |
| Uganda | 2006 | 13.6 | 9.5 | 15.6 | 14.5 | 11.7 | 23.1 | 9.3 | 11.0 | 21.0 | 40.9 | 9.4 | 10.0 | 10.3 | 11.8 | 23.2 |
|  | 2011 | 30.8 | 21.4 | 34.8 | 33.3 | 27.6 | 43.6 | 21.1 | 27.0 | 38.4 | 64.7 | 21.2 | 27.3 | 26.1 | 29.1 | 44.3 |
|  | 2016 | 37.7 | 23.3 | 42.7 | 41.8 | 34.6 | 46.2 | 26.2 | 32.2 | 43.3 | 73.9 | 30.9 | 33.6 | 33.8 | 37.4 | 48.5 |
| Southern |  |  |  |  |  |  |  |  |  |  |  |  |  |  |  |  |
| Lesotho | 2004 | 8.7 | 5.3 | 7.5 | 10.7 | 8.2 | 10.7 | 10.5 | 7.2 | 10.2 | 33.5 | 7.36 | 5.4 | 7.2 | 7.9 | 13.3 |
|  | 2009 | 31.5 | 19.8 | 33.9 | 35.5 | 25.5 | 43.3 | 15.6 | 22.3 | 35.4 | 76.9 | 17.13 | 21.5 | 24.6 | 31.8 | 48.2 |
|  | 2014 | 35.1 | 26.0 | 42.2 | 36.1 | 26.6 | 49.9 | 7.5 | 18.1 | 39.8 | 85.2 | 11.36 | 21.55 | 26.7 | 36.7 | 60.2 |
| Malawi | 2000 | 20.4 | 11.9 | 22.0 | 23.1 | 20.5 | 20.0 | 17.7 | 20.6 | 25.9 | 67.0 |  |  |  |  |  |
|  | 2004 | 26.3 | 15.1 | 28.3 | 29.6 | 20.5 | 20.0 | 22.8 | 24.5 | 37.0 | 72.8 | 19.5 | 21.9 | 23.3 | 28.1 | 36.4 |
|  | 2010 | 35.1 | 18.9 | 37.2 | 40.4 | 24.0 | 37.0 | 32.2 | 33.7 | 39.6 | 64.2 | 30.3 | 33.5 | 34.3 | 36.0 | 40.0 |
|  | 2015 | 45.0 | 26.4 | 48.3 | 50.7 | 42.8 | 54.7 | 42.3 | 40.9 | 52.5 | 83.9 | 40.4 | 41.3 | 41.8 | 44.1 | 55.0 |
| Namibia | 2000 | 20.6 | 15.4 | 25.4 | 21.0 | 14.3 | 29.7 | 9.0 | 13.3 | 25.9 | 47.3 |  |  |  |  |  |
|  | 2007 | 20.8 | 14.8 | 22.4 | 22.6 | 11.3 | 30.8 | 6.8 | 9.0 | 23.0 | 57.0 | 8.0 | 8.2 | 11.9 | 21.1 | 45.4 |
|  | 2013 | 43.3 | 32.7 | 54.2 | 43.5 | 26.9 | 55.9 | 13.1 | 18.7 | 46.5 | 84.1 | 21.2 | 25.1 | 32.7 | 53.3 | 70.2 |
| Zambia | 2002 | 9.5 | 5.8 | 9.5 | 11.1 | 7.4 | 12.5 | 4.5 | 7.5 | 13.0 | 34.2 |  |  |  |  |  |
|  | 2007 | 9.3 | 5.2 | 10.6 | 10.5 | 4.8 | 15.6 | 2.6 | 6.7 | 12.2 | 33.9 | 3.0 | 4.1 | 5.2 | 11.6 | 18.6 |
|  | 2013 | 21.5 | 11.6 | 22.4 | 24.9 | 16.3 | 27.5 | 15.1 | 16.8 | 23.7 | 58.5 | 11.4 | 17.5 | 18.9 | 22.6 | 32.2 |
|  | 2018 | 30.3 | 16.5 | 32.2 | 34.8 | 21.2 | 40.7 | 18.1 | 22.1 | 35.4 | 73.1 | 17.1 | 19.8 | 23.2 | 33.2 | 49.9 |
| Zimbabwe | 1999 | 11.2 | 7.5 | 11.0 | 13.0 | 9.3 | 14.3 | 7.7 | 9.5 | 12.2 | 28.3 |  |  |  |  |  |
|  | 2005 | 15.1 | 9.8 | 15.3 | 17.3 | 10.2 | 22.6 | 10.7 | 8.9 | 17.3 | 44.4 | 9.0 | 8.5 | 10.6 | 17.2 | 24.6 |
|  | 2010 | 19.6 | 11.1 | 22.7 | 21.7 | 15.5 | 26.2 | 10.2 | 13.9 | 19.5 | 61.2 | 12.4 | 15.5 | 14.8 | 20.4 | 30.4 |
|  | 2015 | 27.9 | 18.8 | 31.6 | 30.2 | 15.9 | 47.2 | 3.0 | 11.6 | 29.4 | 76.3 | 11.8 | 14.9 | 14.1 | 32.5 | 53.1 |
